# Mapping of Minimal Motifs of B-Cell Epitopes on Human Zona Pellucida Glycoprotein-3

**DOI:** 10.1155/2012/831010

**Published:** 2011-11-17

**Authors:** Wan-Xiang Xu, Ya-Ping He, Jian Wang, Hai-Ping Tang, Hui-Juan Shi, Xiao-Xi Sun, Chao-Neng Ji, Shao-Hua Gu, Yi Xie

**Affiliations:** ^1^Department of Reproductive Biology, Shanghai Institute of Planned Parenthood Research, Shanghai 200032, China; ^2^Shanghai Jiai Genetic & IVF Center, Obstetrics and Gynecology Hospital, Shanghai Medical College, Fudan University, Shanghai 200011, China; ^3^State Key Laboratory of Genetic Engineering, Institute of Genetics, School of Life Science, Fudan University, Shanghai 200433, China

## Abstract

The human zona pellucida glycoprotein-3 (hZP3) by virtue of its critical role during fertilization has been proposed as a promising candidate antigen to develop a contraceptive vaccine. In this direction, it is imperative to map minimal motifs of the B cell epitopes (BCEs) so as to avoid ZP-specific oophoritogenic T cell epitopes (TCEs) in the ZP3-based immunogens. In this study, based on known results of mapping marmoset and bonnet monkey ZP3 (mstZP3 and bmZP3), two predictable epitopes^23–30  and  301–320^ on hZP3 were first confirmed and five minimal motifs within four epitopes on hZP3 were defined using serum to recombinant hZP3a^22–176^ or hZP3b^177–348^ as well as a biosynthetic peptide strategy. These defined minimal motifs were QPLWLL^23–28^ for hZP3^23–30^, MQVTDD^103–108^ for hZP3^93–110^, EENW^178–181^ for hZP3^172–190^, as well as SNSWF^306–310^ and EGP^313–315^ for hZP3^301–320^, respectively. Furthermore, the antigenicity of two peptides for hZP3^172–187^ and hZP3^301–315^ and specificity of the antibody response to these peptides were also evaluated, which produced high-titer antibodies in immunized animals that were capable of reacting to ZP on human oocytes, r-hZP3b^177–348^ protein, as well as r-hZP3^172–190^, r-hZP3^303–310^, and r-hZP3^313–320^ epitope peptides fused with truncated GST188 protein.

## 1. Introduction

The human zona pellucida (hZP) is an extracellular matrix surrounding eggs, which consists of four sulfated glycoproteins designated as ZP1, ZP2, ZP3, and ZP4 [1, 2]. ZP glycoproteins mediate several critical events during fertilization process such as initial recognition and binding of the spermatozoa to the egg in a species-specific manner, induction of the acrosome reaction in the zona-bound spermatozoon, and prevention of polyspermy [3, 4]. By virtue of their critical role during fertilization, ZP glycoproteins have been proposed as target for developing contraceptive vaccines [5, 6]. Although active immunization with purified or recombinant ZP (r-ZP) proteins induces infertility in various mammalian species, the contraceptive efficacy is invariably associated with either transient alteration or complete loss of ovarian function [7–10]. The observed ovarian pathology following active immunization with ZP antigens may be due to (i) presence of ZP-specific T-cell epitopes (TCE) within a B-cell epitope (BCE), for example peptide corresponding to mouse ZP3 (mZP3) amino acid (aa) residues 330–342 (peptide^330–342^) [11], (ii) autoimmune ovarian disease (AOD) that can be adoptively transferred by the ZP3 peptide-activated CD4^+^ T-cells to naïve recipients but does not occur when only antipeptide antibodies are administered [12], and (iii) the minimal and modified BCE peptide^335–342^ of mZP3, which had no longer a ZP-specific TCE (phenylalanine, a key residue of the “oophoritogenic” TCE, substituted by alanine), induced infertility in mice with eight different haplotypes without any AOD when it was co-linearly synthesized with a foreign “promiscuous” TCE [13].

The above observations clearly suggest a new possible approach of developing BCE peptide-based ZP vaccines for fertility control, which should be devoid of any ZP-specific TCE. Therefore, efforts have been made by several laboratories to delineate infertility-associated BCEs of ZP proteins since a BCE on mZP3 was first identified by Dean et al. [14]. For instance, five BCE peptides^45–64,  93–110,  137–150,  172–190  *and*  334–341^ of hZP3 have been mapped [15–17] and additional two epitopes corresponding to aa residue 23–30 and 301–320 of hZP3 have also been predicted based on the mapping results of marmoset ZP3 (mstZP3) and bonnet monkey (Macaca radiata; bmZP3) proteins [18, 19], because their aa sequences are highly conserved among mstZP3 and hZP3 as well as bmZP3 and hZP3 proteins. To eliminate potential oophoritis-inducing TCE within a mapped longer BCE peptide, the identification of minimal motif of mapped BCEs on hZP3 has been hampered due to the limitation of available mapping methods. So far, only the minimal motif of the BCE on the C-terminus of hZP3 has been identified using rabbit serum [16].

We have reported previously the identification of a minimal motif of hZP4^314–319^ epitope using improved peptide biosynthesis strategy for the first time, where the truncated streptavidin (Stv108) and glutathione S-transferase (GST188) were used as protein carrier, respectively [20]. In addition, the sera against r-hZP3^22–176^ (hZP3a) and r-hZP3^177–348^ (hZP3b) were made, which reacted with human oocyte [21]. Therefore, the main aim of the present study was to map minimal motifs of four linear BCEs on hZP3 with biosynthetic peptides and antibodies against r-hZP3a or r-hZP3b. Moreover, the immunogenicity of two chemically synthesized peptides^171–186  *and*  301–315^ of hZP3, conjugated to keyhole limpet hemocyanin (KLH), has also been evaluated in rabbits.

## 2. Materials and Methods

### 2.1. Plasmids, Antibodies, and Peptides

The plasmids pXXStv-3 and pXXGST-1 were used to express various biosynthetic peptides in *E. coli* [20]. Rabbit sera against r-hZP3a and r-hZP3b prepared as described previously were used for BCE identification and minimal motif mapping [21]. Peptides FSLRLMEENWNAEKRS (P1) and SFSKPSNSWFPVEGP (P2) corresponding to hZP3^172–187^ and hZP3^301–315^ as well as PETQPGPLTLELQIAKDK (P3) corresponding to hZP4^308–325^ were produced on an APEX396 synthesizer by Sangon Co. (Shanghai, China), with more than 90% purity on HPLC. The peptides P1 and P2 were used as antigens to test antibody against P1 or P2 by ELISA, and peptide P3 was used as unrelated control peptide in ELISA.

### 2.2. Other Reagents

All chemicals were purchased from Sangon Co. unless otherwise stated.

### 2.3. Biosynthesis of 6/8mer–20mer Peptides

A set of biosynthetic peptides (numbering P4–P11) corresponding to P4 (QPLWLLQG), P5 (ECQEATLMVMVSKDLPGTGK), P6 (EVGLHECGNSMQVTDDAL), P7 (PIECRYPRQGNVSS), P8 (FSLRLMEENW), P9 (FSLRLMEENWNAEKRSPTF), P10 (SFSKPSNSWFPVEGPADICD), and P11 (RRQPHVMS) of hZP3^23–30,  45–64,  93–110,  137–150,  172–181,  172–190,  301–320  *and*  334–341^ were expressed in *E. coli* as truncated GST188 or Stv108 fusion proteins as described earlier [20]. Similarly, four sets of 35 overlapping 6–8mer peptides (P12–P46) corresponding to P4, P6, P9, and P10 sequences were also made, which overlapped each other by 5–7 residues.

Briefly, the synthesized annealed DNA fragments encoding each P4 to P46 peptides corresponding to the hZP3 cDNA sequence [22], incorporating BamH I and TAA-Sal I cohesive end on their 5′ and 3′ ends, were inserted into the BamH I and Sal I sites downstream of the Stv108 or GST188 gene in pXXStv-3 or pXXGST-1 plasmid. The resultant recombinant plasmids expressing each target short peptide fused with Stv108 or GST188 protein were transformed into the BL21(DE3)(pLysS) *E. coli* strain (Novagen, Inc., Madison, Wis, USA). Each recombinant clone was first grown in 3 mL of Luria Broth (LB) containing 100 *μ*g mL^−1^ ampicillin at 30°C with continuous shaking at 200 rpm overnight. Next day, 60 *μ*L of cell suspension was inoculated in 3 mL of fresh LB and grown until the cell density reached 0.6–0.8 at OD600, and then further grown for 4 h at 42°C to induce expression of the recombinant protein. For screening positive recombinant clones, each cell pellet harvested from induced target clones was first used to run SDS-PAGE gel using the pellet containing Stv108 or GST188 protein expressed by pXXStv-4 or pXXGST-2 plasmid as negative controls, and nucleotide (nt) sequence of all the recombinant clones were subsequently determined (United Gene Holding Ltd., Shanghai). The cell pellets containing each short peptide fusion protein were stored at −20°C.

### 2.4. SDS-PAGE and Western Blotting

Cell pellets obtained from 3 mL culture were boiled in 400 *μ*L of 1x sample loading buffer for 5 min and proteins were resolved by SDS-PAGE under reducing conditions using 15% gels [23]. Gels were either stained with Coomassie brilliant blue G-250 for analyzing the bands of fusion proteins or processed for Western blot by electrotransferring the proteins onto 0.2 *μ*m nitrocellulose membrane (Whatman GmbH, Dossel, Germany) [24]. Complete transfer of proteins was ensured by staining the nitrocellulose membrane with 0.1% (w/v) Ponceau S. Nitrocellulose membrane was subsequently processed for Western blotting using rabbit antisera against r-hZP3a and r-hZP3b (1 : 300 dilution in PBS containing 0.05% Tween 20 and 1% skim milk powder) or immune sera against P1 or P2 (1 : 2000 dilution). Specific antigen-antibody reactions on the membrane were visualized by using goat anti-rabbit IgG conjugated to horseradish peroxidase (HRP) (Proteintech Group, Inc., Chicago, Ill, USA) at 1 : 1000 dilution. The blot was developed by using 3,3′-diaminobenzidine (DAB) (Sigma, Mo, USA) in 50 mM PBS containing 0.05% H_2_O_2_. The reaction was stopped by washing the membrane extensively with MQ water. In some cases, enhanced chemiluminescence was also performed using ECL plus Western blotting detection reagents (GE Healthcare, Buckinghamshire, UK) according to the manufacturer's instructions.

### 2.5. Immunization of Animals

Care and treatment of the animals was based on the standard laboratory animal care protocols approved by the Institutional Animal Care Committee. Six male New Zealand White rabbits were obtained from SIPPR-BK Lab Animal Co., Ltd. (Shanghai, China). The immunization schedule was as follows: six rabbits (number 073–076) weighing 2.0 ± 0.5 kg were immunized intramuscularly with 0.5 mg of chemically synthesized peptide P1 or P2 conjugated with KLH (Sigma) and emulsified in complete Freund's adjuvant (CFA) (Sigma) at multiple sites on the rabbit's back, respectively. The animals were boosted three times intramuscularly with 0.25 mg of same peptide antigen emulsified in incomplete Freund's adjuvant (Sigma) at 2 week intervals. Serum samples from immunized animals were collected 7 days after the third booster, and the antibody titer was assessed by ELISA. Nonimmune sera from two animals (number 077-078) who only received CFA and P3 as unrelated peptide antigen were used as negative controls in the ELISA, respectively.

### 2.6. ELISA and Immunofluorescence

#### 2.6.1. ELISA

The 96-well ELISA plates (Greiner bio-one; Germany) were coated with 50 *μ*L of synthetic peptide P1 or P2 (100 ng per well) overnight at RT. Unbound synthetic peptide was washed off with PBS containing 0.05% Tween 20 (PBST), and sera against P1 or P2 diluted 1 : 50 in blocking buffer (0.01 M PBS containing 5% skim milk powder and 0.05% Tween) were added to the wells (50 *μ*L/well) at two-fold serial dilutions. After incubation for 2 h at RT, plates were washed three times with PBST. To visualize specific peptide-antibody reactions, 50 *μ*L of goat-anti-rabbit IgG conjugated to HRP diluted 1 : 1,000 in PBS was added to wells and incubated for 1 h at RT. All wells were treated with 0.4 mg mL^−1^ o-phenylenediamine and 0.015% (v/v) H_2_O_2_ after washing as before and the reaction was stopped with H_2_SO_4_. Finally, the absorbance was read at 490 nm, according to the manufacturer's instructions (Thermo Fisher Scientific, Pittsburgh, Pa, USA, Product number 34062T) using an ELX 800 Universal Microplate Reader (Bio-TEK Instruments, Inc. Vt, USA). For negative control, the same amount of unrelated P3 synthetic peptide was used for coating the wells. In addition, rabbit preimmune serum was also used as negative control

#### 2.6.2. Immunofluorescence

Experiments using human oocytes was approved by the Institutional Ethics Committee. Human oocytes that had failed to fertilize during in vitro fertilization (IVF) treatments were kindly donated, and a signed written consent for use of oocytes was obtained from all participants. Reactivity of antisera against P1 or P2 with native human ZP was evaluated by an indirect immunofluorescence. Briefly, all oocytes were washed thrice with PBS containing 1 mg mL^−1^ of polyvinylalcohol (PVA), and then fixed in 4% (w/v) paraformaldehyde in 50 *μ*L of PBS for 5 min at RT. Next, the fixed oocytes were washed with PBS-PVA and incubated for 45 min in a blocking solution containing 3% normal goat serum. Further, oocytes were incubated with 1 : 50 dilution of preimmune or immune sera in PBS-PVA for 1 h at 37°C after washing with PBS-PVA. Finally, oocytes were treated with 1 : 500 dilution of Alexa Fluor 568 goat anti-rabbit IgG (Invitrogen, Calif, USA) for 30 min at 37°C, and after washing, the treated oocytes in PBS-PVA were examined under a Nikon TE300 inverted microscope (Nikon Co., Tokyo, Japan).

## 3. Results

### 3.1. Reidentification of Known and Predictable Linear BCEs on hZP3 Protein

**Figure 1:**
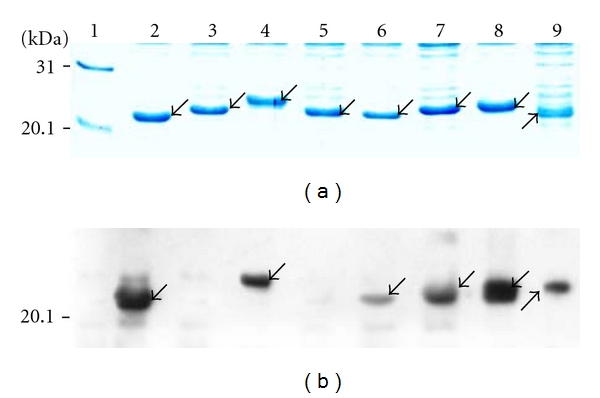
(a) SDS-PAGE analysis of expressed P4–P11 peptides fused with GST188 protein. (b) Western blotting of each BCE peptide using a mixture of sera to r-hZP3a and r-hZP3b. Lane 1, prestained protein marker; 2, lanes 2–9, P4–P11 fusion proteins. Arrows in a-b indicate the bands comprising expressed P4–P11 fusion proteins and their respective bands.

To determine whether those identified (hZP3^45–64,  93–110,  137–150,  172–190  *and*  334–341^) and predicted (hZP3^23–30,  172–181  *and*  301–320^) BCE peptides could be recognized by rabbit sera to r-hZP3a and r-hZP3b, the P4–P11 peptides, which were fused with truncated GST188, were constructed with DNA recombinant technology. As shown in Figure 1(a), P4–P11 fusion proteins were expressed in *E. coli*. Surprisingly, the electrophoretic mobilities of some short peptides fused with truncated GST188 did not seem to match their molecular weight, such as, 20mer P5 protein (Figure 1(a), Lane 3) migrated similarly to that of 14mer P7 protein (Lane 5) than to that of 18mer P6 protein (Lane 4) and so on. Although this aberrant behavior cannot be explained at present, these electrophoretic results should be reliable as their respective nt sequence matched with the aa sequence. Further, expression of the respective fusion protein was not observed in the uninduced clones (data not shown).

Six peptides (P4, P6, P8, P9, P10, and P11) of hZP3 could be recognized by antiserum against r-hZP3a or r-hZP3b, but P5 and P7 failed to react with both the immune sera in Western blot (Figure 1(b)). The results confirmed three BCE peptides (P6, P9, and P11) of hZP3 out of five BCE peptides identified previously. All three predicted BCE peptides also reacted with antibodies against r-hZPa/r-hZP3b. In a word, this study showed that there at least were five BCE peptides on hZP3 protein.

### 3.2. Mapping of Minimal Motifs on P4, P6, P9, and P10 Epitope Peptides

**Figure 2:**
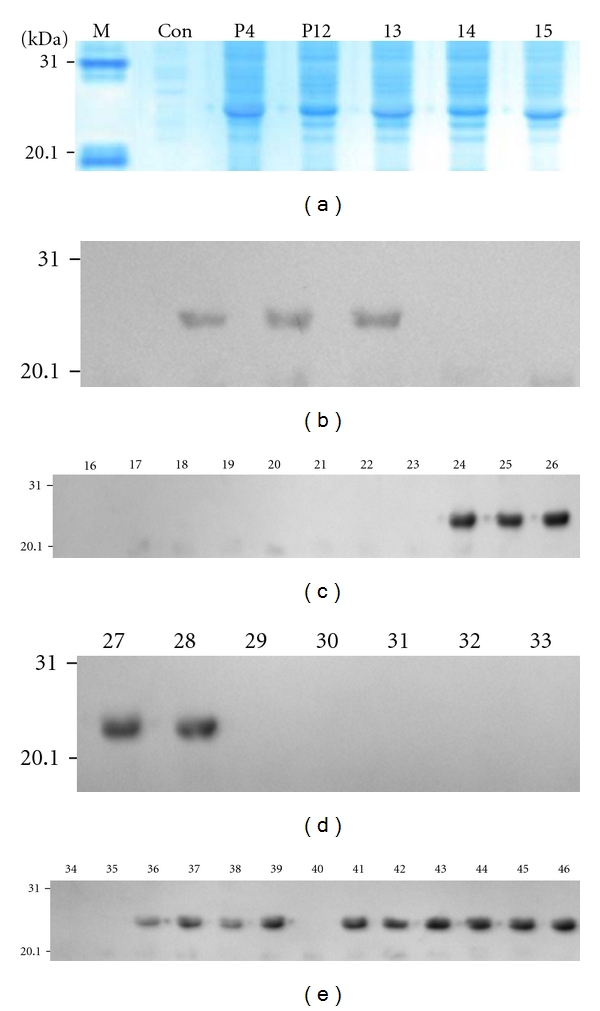
(a) SDS-PAGE analysis of expressed P4 and P12–P15 fusion proteins. (b)–(e) Western blotting of GST188-8mer peptides from P4, P6, P9, and P10 peptides detected using sera to r-hZP3a or r-hZP3b. Note: M, prestained protein marker; Con, uninduced total cell proteins as negative control; P4 and P12–46, total cell proteins of expressed P4; P12–P46 8mer peptide fusion proteins.

**Figure 3:**
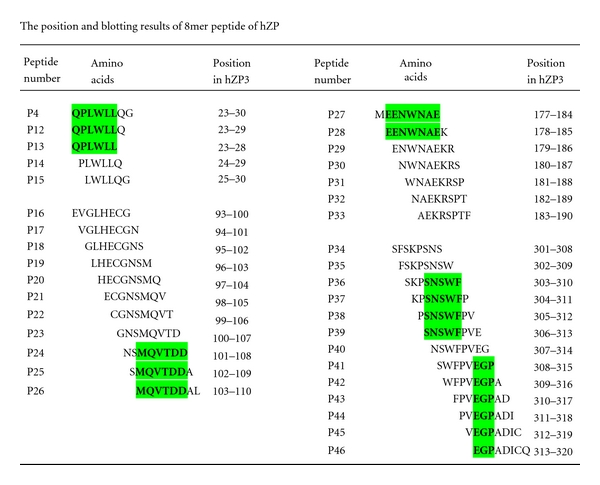
The synthetic 6/8mer peptide sequences from hZP3 protein. The **green** highlight indicates the common sequence recognized by antibodies to r-hZP3a or r-hZP3b in P4, P6, P9, and P10 fusion proteins.

Based on initial mapping result, the peptides P4, P6, P9, and P10 were selected to identify their minimal motifs with sera to r-hZP3a or r-hZP3b, except P11 that its motif has been known [16]. Four sets of thirty-seven 6–8mer peptides with an overlap of 5–7 aa residues for P4, P6, P9, and P10 peptide were constructed and used to map minimal motifs in this study. All P4 and P12 to P46 peptides were expressed in *E. coli* at a higher level (Figure 2(a), others not shown). As shown in Figure 2, three (b-c), two (d), as well as four and six (e) overlapping 6–8mer peptides fused with GST188 were recognized by serum against r-hZP3a or r-hZP3b. These 36 constructs shared sequences of 6 aa (P4 and P6), 7 aa (P9), as well as 5 aa and 3 aa (P10), respectively (Figure 3). Thus, their minimal binding motifs were localized to residues QPLWLL^23–28^, MQVTDD^103–108^, EENWNAE^178–184^, SNSWF^306–310^, and EGP^313–315^ on the epitope peptides P4, P6, P9, and P10, of which two minimal motifs on peptide P10 were identified, suggesting that there were two nested BCEs on it.

Additionally, we prejudged that the peptide P8 of hZP3^172–181^ should be recognized by serum to r-hZP3b, because the mapped epitope sequence^171–180^ of mZP3 [25] is 100% conserved among mZP3 and hZP3 at the amino acid level. However, they did not generate a blotted brown band at the position of Stv108-P8 fusion protein when initially using DAB coloration due to its relatively lower sensitivity (result not shown). Therefore, a set of P4–P11 peptides fused with GST188 was again constructed, which were suited to chemoluminescence detection on the blotted membrane and then identified when using high-sensitivity ECL plus Western blotting detection reagents. As a result, the peptides P8 and P9 were recognized by serum to r-hZP3b (Figure 1(b), lanes 6-7). Thus, the minimal motif^178–181^ of peptide P9 was finally defined according to the shared sequences of 4 aa residues (EENW) between P8 and the minimal motif of hZP3^178–184^ mapped initially.

### 3.3. Antigenicity of Peptides P1 and P2

**Figure 4:**
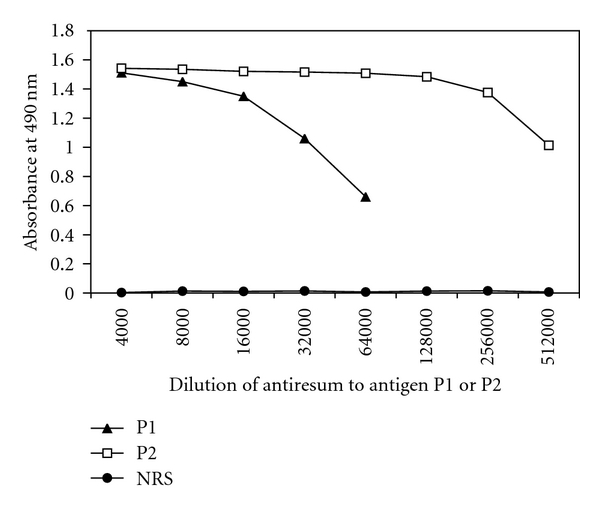
Comparison antibody titers to synthetic P1 and P2 of hZP3. The antibody levels were determined by ELISA using synthetic P1 (▲) and P2 (□) peptide as antigens. Normal rabbit serum (●) and or P3 (not shown) were used as negative controls. Titers were determined based on the highest dilution of the sample that generated OD greater 0.2.

To investigate and compare antigenicity of peptides P1 and P2, each two rabbits (number 073-074 and number 075-076) were immunized with synthetic peptide P1 or P2 antigen in FCA, which were conjugated with KLH. As shown in Figure 4, each peptide antigen all elicited higher antibody responses against peptide P1 or P2 in immunized animals, of which the rabbit (number 073) immunized with P1 antigen showed antibody titers of 6.4 × 10^4^, whereas that level in the rabbit (number 076) immunized with P2 antigen reached approximately 5.1 × 10^5^ in ELISA assay.

### 3.4. Specificity of Antibodies against Peptides P1 and P2

**Figure 5:**
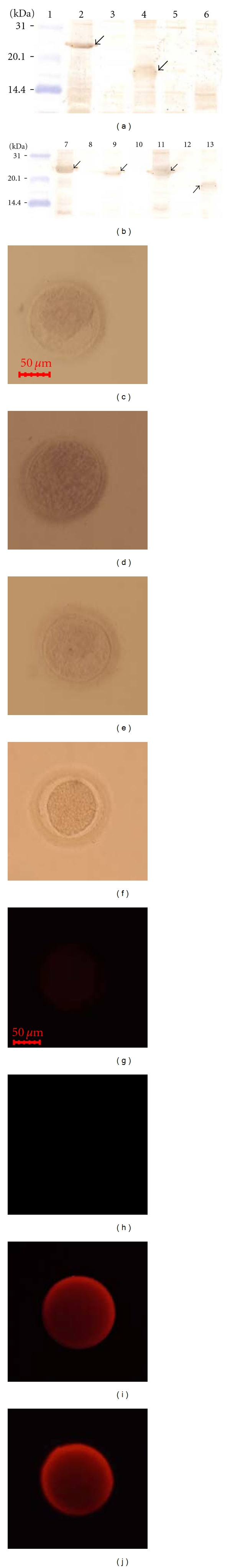
(a-b) Western blotting with sera to P1 or P2. Lane 1, prestained protein marker; Lanes 2 and 3, induced and uninduced GST-hZP3^172–190^; Lanes 4 and 13, induced hZP3b^177–348^; Lanes 5 and 6, induced Stv108 and GST188; Lanes 7 and 8, induced and uninduced GST-hZP3^301–320^; Lanes 9 and 10, induced and un-induced GST-P37; Lanes 11 and 12, induced and un-induced GST-P44. (c–f) matching light images for the human oocytes imaged by immunofluorescence in panels g–j. (g–j) reactivity of rabbit sera to P1 or P2 with human oocyte by indirect immunofluorescence. Representative immunofluorescence patterns are shown for (g) preimmune serum; (h) immune serum only received CFA (i-j) immune serum to P1 or P2.

The rabbit sera against P1 or P2 reacted to not only synthetic peptide P1 or P2 in ELISA, but also to P9 (hZP3^172–190^) or P10 (hZP3^301–320^)-GST188 fusion protein (Figures 5(a) and 5(b), Lane 2 and 7) as well as native human ZP in indirect immunofluorescence (Figures 5(i) and 5(j)). As shown in Figure 5(a), the serum against P1 did not react with Stv108 and GST188 carrier proteins (Lanes 5-6), so does the serum against P2 (data not shown), suggesting there was no cross-reactive antibodies with Stv108 and GST188 proteins in their immune sera. In addition, the serum to P2 reacted to 8mer peptides P37 and P44 containing a mapped minimal motif SNFWF or EGP (Figure 5(b), Lanes 9 and 11), confirming our above mapping result, that is, there were two overlapping BCEs within the epitope peptide of hZP3^301–320^ (Figure 2(e) and Figure 5(b)). As shown in Figure 5, the rabbit serum to P1 or P2 diluted to 1 : 50 showed a strong positive reaction with the human ZP (I-J), and red fluorescence was absent when the oocytes were treated with preimmune serum (g-h). These results showed that production of specific immunoglobulin reactive with P1 or P2 epitope peptide and with human ZP was elicited by P1 or P2 peptide in immunized rabbits, respectively.

## 4. Discussion

The hZP3 protein has been an interesting target antigen for the development of a contraceptive peptide vaccine. Two BCEs^137–150  and  334–341^ on hZP3 were first identified with serum to each synthetic peptide based on computer prediction or sequence comparison of a known BCE sequence of mZP3 with other ZP3 protein in many mammals [15, 16]. The former 14 mer peptide^137–150^ (P7) of hZP3, however, was not recognized by both sera to r-hZP3 in ELISA [17] and serum to r-hZP3a in Western blotting (Figure 1(b), Lane 5), suggesting that it might not be a self-epitope peptide of hZP3, although it could elicit antibody capable of binding to native hZP and its antiserum might be used as a marker for the identification of hZP3 protein [15]. For the latter 8 mer peptide^334–341^ (P11) that elicited antibodies reacting to human ZP in transgenic mice [16], although it failed to be identified by serum to r-hZP3 in the study on epitope mapping of hZP3^22–360  ^ [17], the result that peptide P11 could be recognized by serum to r-hZP3b in this study (Figure 1(b), lane 9) suggested that it should be an epitope of hZP3. As well, the 20 mer peptide^45–64  ^ (P5) of hZP3 mapped by serum to r-hZP3 [17], it could not react to serum to r-hZP3b in this study (Figure 1(b), Lane 3). At present, we cannot explain these distinct mapping results, including the above-mentioned 8mer peptide^334–341^, because both groups employed different antisera (against r-hZP3^22–460  ^ and r-hZP3a^22–176^, resp.).

The antigenicity and immunogenicity of hZP3^172–190^ [17], mstZP3^301–320^ [18, 26], and bmZP3^300–322^ [27] were previously reported. These synthetic peptides elicited antibodies capable of reacting to native mstZP, bmZP, and hZP, respectively. The peptide sequence^301–320^ is highly conserved between mstZP3 and bmZP3 sequences. The mst-serum tomstZP3^301–320^ linked to a promiscuous helper TCE from tetanus toxoid showed up to 60% of inhibition in human sperm-zona binding in vitro [26]. Conversely, the bm-serum to bmZP3^300–322^ conjugated to diphtheria toxoid failed to show any significant decrease in sperm-zona binding in hemizona assay [27]. The reasons for this discordance are not clear and more research is required to explain these findings. In the present study, because there is difference of one residue at the position^305^ of hZP3^301–320^ (P), mstZP3^301–320^ (A), and bmZP3^301–320^ (S), its antigenicity and specificity was preliminarily evaluated using synthetic peptide P2 conjugated with KLH, and was compared with peptide P1. As shown in Figures 4 and 5, peptides P1 and P2 elicited high-titer antibodies reacting with native hZP and r-hZP3b peptide expressed in *E. coli*, but the antigenicity of the latter was significantly greater than that of the former. For the antigenic diversity of peptides P1 and P2, one possible explanation might be because there were antibodies against two overlapping BCEs (NSNWF^306–310^ and EGP^313–315^) within the peptide P2 sequence. As for the efficacy of each antiserum to inhibit human sperm-zona binding, it remains to be further evaluated by carrying out competitive hemizona or sperm-egg binding assays.

The serum to a synthetic peptide was often used to map minimal motif of identified BCE peptide [16, 28]. To further check the specificity of peptides P1 and P2 as antigen, the Western blotting was carried out with two sets of 8 mer peptides (P27~P33 and P34~P46). Surprisingly, the rabbit serum to P1 not only could recognize peptides P28 and P29, but also reacted with peptides P30 and P31 that were not recognized by rabbit serum to r-hZP3b, whereas the serum to P2 only recognized peptides P42 and P43, but did not recognize other peptides (P43–P46) reacted with serum to r-hZP3b (data not shown). The data suggested that their BCE motifs generated a “drifting” phenomenon when using serum against synthetic peptide P1 or P2 to map minimal motif compared with the result identified with serum to r-hZP3b, that is, the minimal motifs mapped with serum to a synthetic peptide and to a native or r-protein might be different sometimes.

The synthetic peptides method [29–31] has been often used to delineate linear BCEs on a protein; however, the number of mapped BCE always was less or some BCEs were missing when using serum to r-protein and ELISA. For instances the following hold. (1) Only 3 BCEs on hZP3, not including a BCE on its C-terminus transmembrane- like domain, were mapped with serum to CHO-expressed r-hZP3^22–460^ [17] compared with the result of mapped 6 BCEs in this study. (2) It could not be defined how many BCEs there were in six of potential reactive neighboring 15mer-peptides (P5–P8, P11-P12, P14–P18, P31–P33, P38-P39, and P43-P44) in the epitope mapping of brushtail possum (bp) ZP2^40–634^ with sera to r-bpZP2^40–311/305–634^ [32], which might be one of causes why several BCE peptides could be missed or could not be defined in above two epitope mapping studies using synthetic peptide library. As we know, there may be only one BCE on mapped two neighboring overlapping peptides because they share a common sequence [33], but there may also be two or three BCEs according to our other epitope mapping results of E6, E7, and L1 proteins from HPV type 58 virus (data not shown). It obviously is a drawback to employ this method to map all BCEs on a protein, because it could not be used to carry out minimal motif identification of each reactive neighboring peptide with serum to r-protein, which is a way of answering how many BCEs there are on them.

Some studies on expression of single 4–12mer BCE peptides fused with Stv118 core protein [34] and epitope mapping with several purified short peptides fused with GST226 [35, 36], suggested the possibility of using biosynthetic peptides to map linear BCEs and their minimal motifs on a protein. However, not like the above studies using mAb, chicken sera to SARS-CoV, and SARS convalescent sera, it needs to solve a key problem to employ serum against r-protein, that is, how to avoid interfering of antibodies against some strong antigens from *E. coli* on distinguishing target blotted bands. Because any r-protein used as immunogen always contains a little bacteria proteins that could not be completely removed when purified at laboratory level. At present, this problem has been solved through using a truncated GST188 or Stv108 as carrier of short peptide expression. As showed in Figure 1, the blotted bands of the GST188-short peptides were to be located in a weak antigenic area of bacterial proteins on blotted membrane, which avoided two blotted bands of bacterial protein with 21 kDa and 31 kDa bands. Obviously, besides simple, cheap, reliable, and adaptable merits mentioned in our previous work [20], the present study showed another two distinct advantages of our improved biosynthetic peptide strategy: (1) it permits using serum against bacteria-expressed protein to map BCEs and their minimal motifs on the target protein; (2) the 8mer–20mer peptide fusion proteins expressed by pXXGST-1 or pXXStv-3 plasmid could be used in Western blotting without purifying. In addition, the smallest binding motif of 3 residues for antibodies can be defined with biosynthetic peptides (Figure 2(e) and Figure 3), whereas the length mapped with synthetic peptides method was 4-5 residues [37].

In summary, this study is the first to utilize biosynthetic peptides and sera against r-hZP3a and/or r-hZP3b to map BCEs and their minimal motifs on hZP3 protein. The identification of five minimal motifs within four epitopes on hZP3 will help in developing contraceptive vaccines of multiepitope ZP peptides without ZP-specific TCE activities which may result in ovarian dysfunction for human use in future, and those defined BCE minimal motifs of hZP3 would be also used as specific probes to detect whether there are self- ZP antibodies to each BCE in the sera from patients with infertile and/or premature ovarian failure. Furthermore, the data presented here again clearly suggest that the biosynthetic peptide strategy employed in this study could be used to map all BCEs and precise linear epitopes on other entire ZP proteins in any species.
